# FGF2 Translationally Induced by Hypoxia Is Involved in Negative and Positive Feedback Loops with HIF-1α

**DOI:** 10.1371/journal.pone.0003078

**Published:** 2008-08-27

**Authors:** Caroline Conte, Elodie Riant, Céline Toutain, Françoise Pujol, Jean-François Arnal, Françoise Lenfant, Anne-Catherine Prats

**Affiliations:** 1 Institut National de la Santé et de la Recherche Médicale (INSERM), U858, Toulouse, France; 2 Université Toulouse III Paul Sabatier, Institut de Médecine Moléculaire de Rangueil, IFR31, Toulouse, Francem; 3 Centre Hospitalier Universitaire de Toulouse, Toulouse, France; Victor Chang Cardiac Research Institute, Australia

## Abstract

**Background:**

Fibroblast growth factor 2 (FGF2) is a major angiogenic factor involved in angiogenesis and arteriogenesis, however the regulation of its expression during these processes is poorly documented. FGF2 mRNA contains an internal ribosome entry site (IRES), a translational regulator expected to allow mRNA expression during cellular stress.

**Methodology/Principal Findings:**

In the present study, we have developed a skin ischemia model in transgenic mice expressing a reporter transgene under the control of the FGF2 IRES. The results reveal that FGF2 is induced at the protein level during ischemia, concomitant with HIF-1α induction and a decrease in FGF2 mRNA. In addition, the FGF2 IRES is strongly activated under these ischemic conditions associated with hypoxia, whereas cap-dependent translation is repressed by 4E-BP hypophosphorylation. We also show that up-regulation of FGF2 protein expression in response to hypoxia correlates with the increase of FGF2 IRES activity *in vitro*, in human retinoblasts 911. The use of siRNAs targeting HIF or FGF2 indicates that FGF2 and HIF-1α reciprocally regulate their expression/accumulation, by a negative feedback loop in early hypoxia, followed by a positive feedback loop in late hypoxia.

**Conclusion/Significance:**

FGF2 expression is up-regulated *in vivo* and *in vitro* in response to hypoxia. Strikingly, this up-regulation is not transcriptional. It seems to occur by an IRES-dependent mechanism, revealing new mechanistic aspects of the hypoxic response. In addition, our data show that FGF2 interacts with HIF-1α in a unique crosstalk, with distinct stages in early and late hypoxia. These data reveal the physiological importance of IRES-dependent translation during hypoxic stress and underline the complexity of the cellular response to hypoxia, suggesting a novel role of FGF2 in the regulation of HIF-1α during the induction of angiogenesis.

## Introduction

The establishment of a stable and functional blood vessel network is a complex process requiring several angiogenic factors to stimulate vessel sprouting and remodeling from the primitive vascular network. Fibroblast growth factor 2 (FGF2) is one of the major regulators of blood vessel formation, involved in angiogenesis as well as in arteriogenesis. However the regulation of its expression during these processes remains to be deciphered [1].

Hypoxia is a major pathophysiological trigger of angiogenesis. In solid tumours, the angiogenic switch responsible for tumour development is induced by hypoxia [2]. In cardiovascular diseases, ischemia corresponds to a shortage of the blood supply, resulting in tissue damage because of the lack of oxygen and nutrients. In ischemic conditions, hypoxia generates a process of revascularization involving both angiogenesis and arteriogenesis [3]. The response to hypoxic stress generates a transcriptional response mediated by hypoxia-induced factor 1 (HIF-1), whose α subunit is stabilized in the absence of oxygen [2]. However, hypoxia also generates the blockade of cellular mRNA translation by impairing the classical cap-dependent mechanism of translation initiation. Translational repression by hypoxia mainly occurs through modulating the activity of two kinases, mTOR and PERK. Inactivation of mTOR results in hypophosphorylation of eIF4E-binding proteins (4E-BP), which increases their affinity for the cap-binding protein eIF-4E and inhibits cap-dependent translation by sequestering eIF-4E [4]. PERK activation by hypoxia, mediated by the unfolded protein response, is responsible for translation inhibition by phosphorylating the translation initiation factor eIF2-α [5].

Translation of specific mRNAs during hypoxia thus requires alternative mechanisms, such as translation initiation mediated by internal ribosome entry sites (IRESs). IRESs are RNA structural elements present in the 5′ non-translated regions of a small number of mRNAs, allowing translation to occur in conditions of stress [6]–[10]. Interestingly, IRESs are present in mRNAs coding for several angiogenic factors, as well as in the HIF-1α mRNA itself [11]. While the transcriptional response to hypoxia is fully documented, little information is presently available, in particular *in vivo*, about the mechanisms regulating IRES-mediated mRNA translation in response to hypoxic stress.

The regulation of FGF2 expression has been mainly described as post-transcriptional: indeed 90% of the FGF2 mRNA, which is transcribed from a single promoter, consists of non-translated or alternatively translated regions [12]. Alternative initiation codons allow for expression of several FGF2 isoforms in human as well as in rodents [13]. This process is crucial for the fate of FGF2, as these isoforms have different localizations and functions [14]. The small 18 kDa isoforms, initiated at an AUG codon, is responsible for the FGF receptor-mediated auto- paracrine activity of FGF2. The high molecular weight isoforms (22, 22.5, 24 and 34 kDa in human, versus 21–22 kDa in mouse), are initiated at in frame, upstream CUG codons, localized in the nucleus and possess intracrine activities [15], [16]. Furthermore, translation of the FGF2 mRNA is under the control of an internal ribosome entry site (IRES) [17]. The FGF2 IRES is the first cellular IRES to have been studied *in vivo*, with transgenic mice expressing bioluminescent, bicistronic transgenes. Previous studies have revealed a strong tissue specificity of the FGF2 IRES in transgenic mice [18]. Interestingly, the IRES mediates FGF2 up-regulation in response to hyperglycemia in the aorta of diabetic mice [19].

In the present study, we investigated the mechanisms regulating FGF2 *in vivo* and *in vitro* in response to hypoxia. We developed a skin ischemia model, using the transgenic mice described above, to analyze endogenous FGF2 expression at the mRNA and protein levels, as well as study the regulation of activity of the FGF2 IRES. Our results revealed that FGF2 is induced at the protein level, concomitant with a decrease in FGF2 mRNA levels, during ischemia. In addition, the FGF2 IRES was strongly activated under hypoxic conditions, whereas cap-dependent translation was repressed by 4E-BP hypophosphorylation. FGF2 expression was also analyzed in human retinoblasts 911, showing that up-regulation of FGF2 protein, but not mRNA, correlates with an increase in activity of the FGF2 IRES *in vitro*. The use of siRNAs targeting HIF or FGF2 indicated that FGF2 and HIF-1α reciprocally regulate their expression/accumulation by a negative, then a positive feedback loop, during early and late hypoxia, respectively.

## Results

### Cutaneous ischemia-induced revascularization in double luciferase transgenic mice

**Figure 1:**
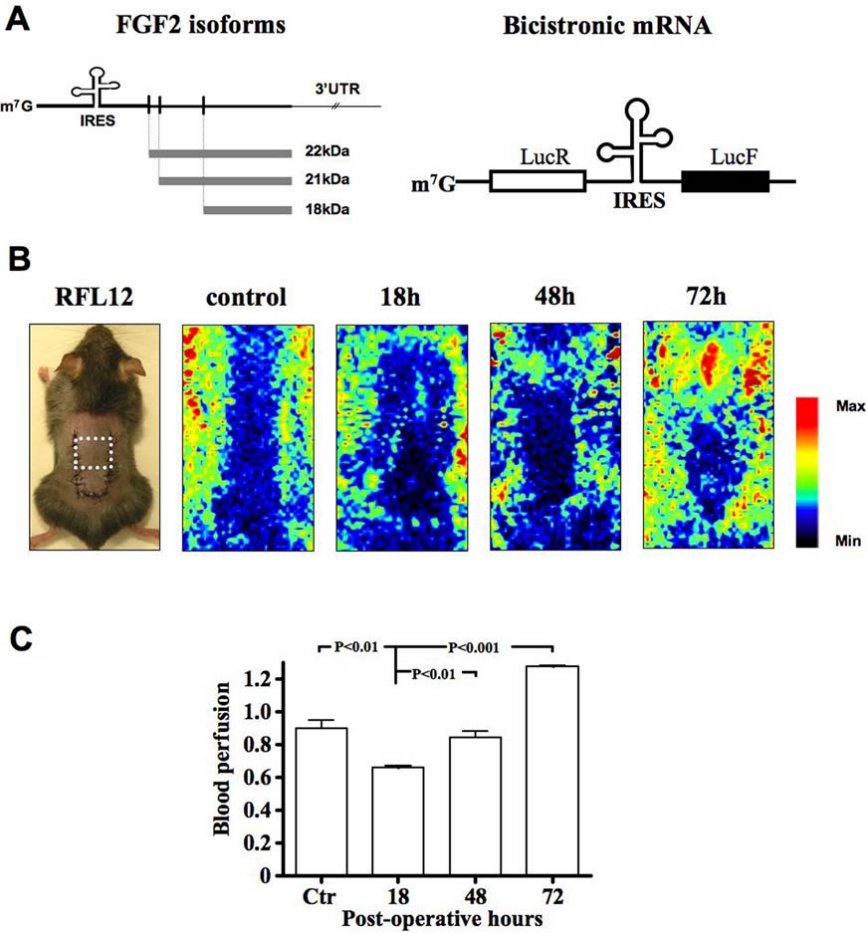
Ischemia induced by a dorsal skin flap model in IRES FGF2-Luc transgenic mice. A. Representation of FGF2 mRNA and mouse protein isoforms (left panel) and of the bicistronic mRNA expressed by the RFL12 transgenic mice (right panel). This bicistronic cassette expresses, under control of the CMV (Cytomegalovirus) promoter, LucR and LucF reporter genes in a cap- or FGF2 IRES-dependent manner, respectively [18]. B. Ischemia induction using a skin flap model modified from Ceradini *et al* [26] and representative Laser Doppler analysis performed 18, 48 and 72 hours after surgery. A U-shaped peninsular skin incision was created on the dorsal surface of 8-week old female RFL12 mice. The two vascular pedicles arising from the lateral thoracic arteries were sectioned. To avoid necrotic tissues, the study of gene expression was performed on the proximal part of the skin flap indicated by the white square. The color scale illustrates blood flow variations from maximal (red) to minimal perfusion (dark blue). C. Quantification of laser Doppler analysis. Ctr corresponds to non-operated mice. Results represent mean±SE on at least 3 mice per post-operative time.

To investigate the impact of ischemia on FGF2 expression and IRES activity, cutaneous ischemia was induced, using a skin flap model, in RFL12 transgenic mice expressing a bicistronic mRNA encoding both *Renilla* luciferase (LucR) and *Firefly* luciferase (LucF) separated by the FGF2 IRES (Fig 1A, right) [18]. In such a transgene, LucR expression is cap-dependent whereas LucF is IRES-dependent. A value of IRES activity may be deduced from the LucF/LucR ratio.

Color laser Doppler analysis showed the induction of a reproducible ischemic gradient 18 h after surgery that ultimately led to blood reperfusion at 72 h, progressing slowly from the top of the skin flap (Fig 1B and 1C).

### FGF2 protein expression is induced by ischemia

**Figure 2:**
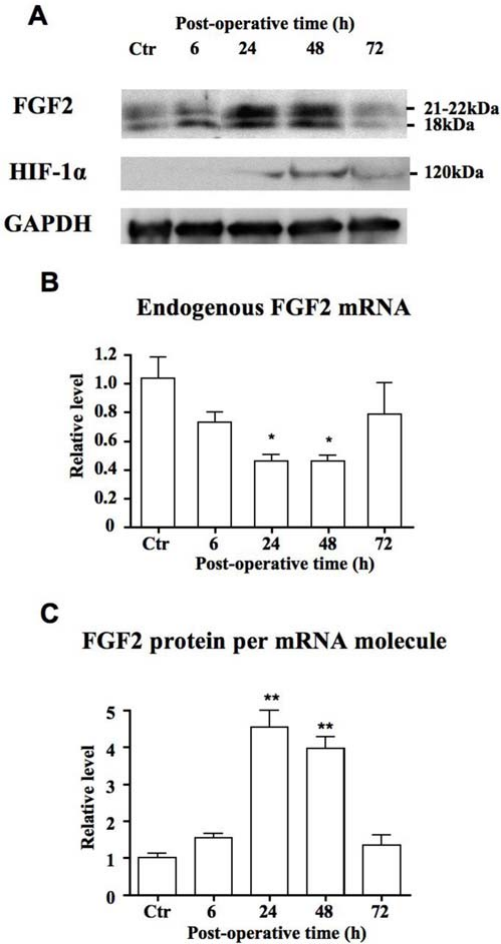
Endogenous FGF2 and HIF-1α expression in ischemic skin extracts. A. Representative Western blot analyses of FGF2 and HIF-1α at the indicated post-operative time. The molecular weights of FGF2 mouse isoforms are indicated. B. Levels of endogenous FGF2 mRNA determined by RT-qPCR analysis. Results are expressed relative to the level from control mice and represent mean±SE (n = 5 mice per post-operative time). *P<0.05 vs. control. C. Quantification of total FGF2 detected by Western blot analysis after normalization to GAPDH and to endogenous mRNA level. **P<0.001 vs. control.

We then analyzed the levels of FGF2 mRNA and proteins in the proximal part of the ischemic skin flap. FGF2 protein levels significantly increased 24 h–48 h after surgery, concomitant with an increase in HIF-1α, indicating that ischemic tissues are submitted to hypoxia (Fig 2A). Interestingly, FGF2 protein induction was already observed 6 h after surgery, whereas HIF-1α was not yet detectable at that time point. In contrast to FGF2 protein levels, FGF2 mRNA levels strongly decreased under ischemic conditions (Fig 2B). These results show that FGF-2 expression is not activated at the transcriptional level in response to ischemia-induced hypoxia and strongly suggests that it could be up-regulated at the translational level. According to the ratio of FGF2 protein expression calibrated to the number of mRNA molecules, FGF2 mRNA translation might be activated four fold after 24–48 h of ischemia (Fig. 2C).

### Ischemia activates FGF2 IRES-dependent translation in vivo

**Figure 3:**
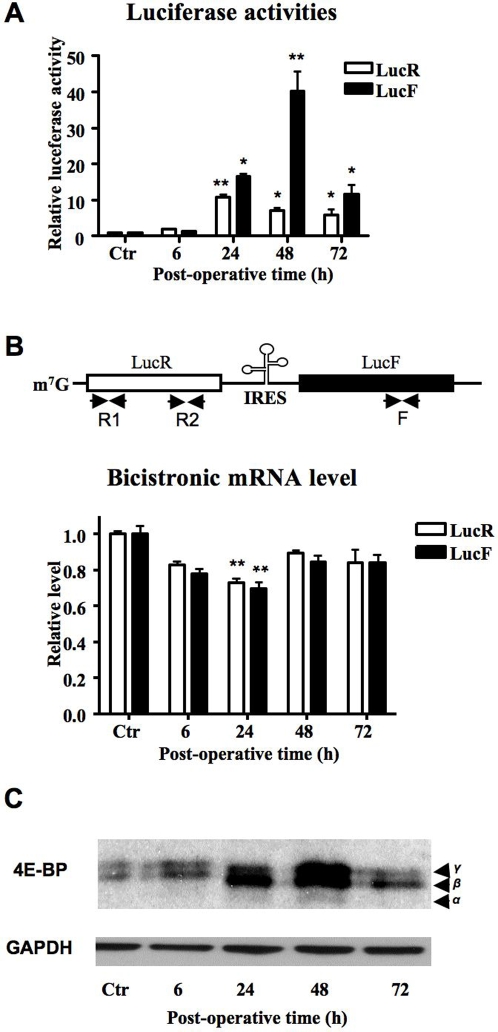
Translational regulation of FGF2 expression in hypoxic conditions *in vivo*. A. Analysis of IRES activity after surgery. Luciferase activities are expressed relative to control mice. LucR and LucF reflect cap- and IRES dependent translation, respectively. The graphic shows the mean±SE (n = 5 mice). * *P*<0.01 and ** *P*<0.001 vs. control mice. B. RT-qPCR quantification of LucR and LucF mRNAs. *Top*. Localization of the primers used for the quantification of LucR and LucF mRNA. *Bottom*. The graphic shows the mean±SE (n = 5 mice). * *P*<0.01 and ** *P*<0.001 vs. control mice. C. Western blot analysis of 4E-BP in ischemic skin extracts at the indicated post-operative time. Positions of the three electrophoretically distinct forms of 4E-BP1 (α–γ in order of increasing phosphorylation) are indicated.

It has been reported that global translation of cellular mRNAs by the classical cap-dependent mechanism is blocked under hypoxic conditions [20]. However several angiogenic growth factor mRNAs, including FGF2, contain IRESs that permit mRNA translation despite the blockade of cap-dependent translation [11], [21]. Therefore, the transgenic mouse model RFL12 enabled us to measure the level of FGF2 IRES-dependent translation in ischemic skin [18]. To study the response of the FGF2 IRES, we measured LucR and LucF activities in the proximal part of the skin flap of the RFL12 mice. A 15 fold increase of LucF activity (IRES-dependent) was observed 24 h after surgery, culminating at a 40 fold increase after 48 h (Fig. 3A). At the same time, cap-dependent LucR activity increased up to 10 fold at 24 h but decreased by 48 h.

Bicistronic mRNA integrity was measured by RT qPCR. In order to check that the observed increase of LucF was not due to the presence of a cryptic promoter or a splicing event that gives rise to monocistronic LucF mRNA, we quantified the transgenic mRNA by using LucR- and LucF-specific probes (Fig. 3B). This method permits detection of the bicistronic and any putative monocistronic transcripts, as shown in previous reports using the same transgenic mouse line as well as in other animal and cellular models [7], [19], [22]–[24]. In such an analysis, the presence of an internal promoter or a splice site would be revealed by the presence of larger amounts of LucF mRNA compared to LucR mRNA [7]. In our experiments, both LucR and LucF mRNAs were always present in equal amounts, indicating that the mRNA is bicistronic (Fig. 3B). RT qPCR analysis of the bicistronic mRNA also showed that its level significantly decreases in ischemic skin, demonstrating that enhancement of LucF as well as LucR activities results from a post-transcriptional event.

In order to know the status of cap-dependent translation under our ischemic conditions, we analysed 4E-BP1 expression and phosphorylation. Hypophosphorylated 4E-BP1 avidly binds to eIF4E and blocks cap-dependent translation initiation, whereas 4E-BP1 hyperphosphorylation abrogates this interaction [25]. Western blot experiments performed on protein extracts of ischemic skin showed an increase of 4E-BP1 under hypoxic conditions (Figure 3C). In addition, the phosphorylation state of 4E-BP1 is modified. Thus the relative level of hypophosphorylated α and β isoforms of 4E-BP1 increased 24 h and 48 h after surgery (Figure 3C). These results indicated that cap-dependent translation is repressed in ischemic skin through the sequestration of eIF4E by its inhibitor 4E-BP1.

These data led us to conclude that FGF2 IRES-dependent translation is strongly activated *in vivo* under ischemic/hypoxic conditions, where cap-dependent translation is repressed. Our findings suggest that the induction of FGF2, observed in Fig 2A, occurs via an IRES-dependent mechanism [26].

### FGF2 expression is translationally induced in response to hypoxia *in vitro*

**Figure 4:**
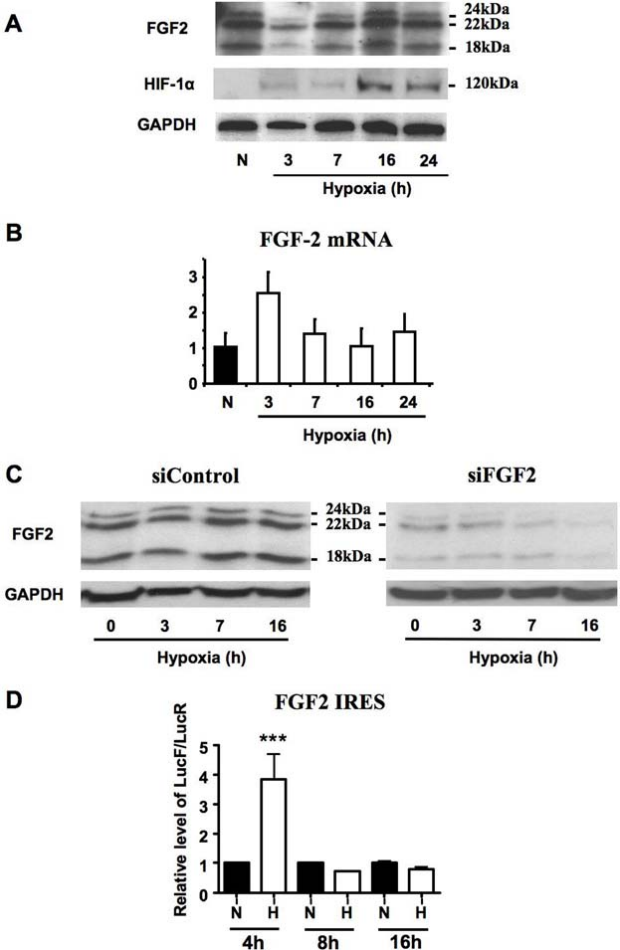
Activation of FGF2 IRES-dependent expression by hypoxia in vitro. A. Representative Western blot analysis of FGF-2 isoforms and HIF-1α in 911 retinoblasts grown in normoxic (N) and hypoxic (H) conditions for the indicated time periods. B. RT-qPCR quantification of FGF2 mRNAs in 911 retinoblasts grown in normoxic (N) and hypoxic (H) conditions. Results are expressed relative to the level of the normoxic value and represent mean±SE (n = 3). C. Accumulation of FGF2 protein during hypoxia in conditions of gene silencing. 911 cells were transfected with FGF2 targeted siRNA (siFGF2) and FGF2 protein contents measured in hypoxic conditions. SiControl corresponds to a control scrambled siRNA. D. Activity of FGF2 IRES measured in 911 retinoblasts expressing the bicistronic construct with the FGF2 IRES in normoxic (N) and hypoxic (H) conditions during 4, 8 and 16 hours. Results are expressed relative to the level of the corresponding normoxic value and represent mean±SE (n = 4). ***P<0.001 vs. the corresponding normoxic condition.

Expression of FGF2 and HIF-1α was studied in 911 human retinoblasts submitted to hypoxia for different periods of time. As shown in Figure 4A and 4B, FGF2 protein expression dropped after 3 hours of hypoxia, began to increase by 7 h and reached a peak at 16 h. In contrast to FGF2 protein levels, the level of FGF2 mRNA increased initially during hypoxia but returned to the basal level at 7 h and later time points. HIF-1α protein was already detectable after 3 hours of hypoxia, but strongly increased at 16 h, coinciding with the peak of FGF2 expression.

The increase in FGF2 protein levels during hypoxia may be due to an increase in protein stability and/or translation. To analyze FGF2 protein accumulation during hypoxia, we used an siRNA knock down approach. FGF2 protein accumulation was measured in 911 cells treated with a siRNA against FGF2 and submitted to hypoxia (Fig. 4C). Under such conditions, FGF2 protein levels progressively decreased and were hardly detectable after 16 h of hypoxia, whereas treatment by the control siRNA led to an accumulation of FGF2. This clearly showed that FGF2 is not stabilized by hypoxia and that protein stabilization can not be responsible for the increase of FGF2 protein levels that culminates at 16 h (Fig. 4A). These data indicated that FGF2 induction from 7 h to 24 h most probably results from an increase in FGF2 mRNA translation and not through effects on FGF2 protein stabilization.

To evaluate the level of IRES activity in response to hypoxia, 911 human retinoblasts were stably transfected with the bicistronic construct described in Fig. 1. Measured luciferase activities indicated that the FGF2 IRES is transiently activated after 4 hours of severe hypoxia in these cells (Fig. 4D).

Taken together, these results suggested that FGF2 expression is induced translationally in response to severe hypoxia and that this induction is presumably mediated by the FGF2 IRES.

### FGF2 and HIF-1α interact in negative and positive feedback loops during hypoxia

**Figure 5:**
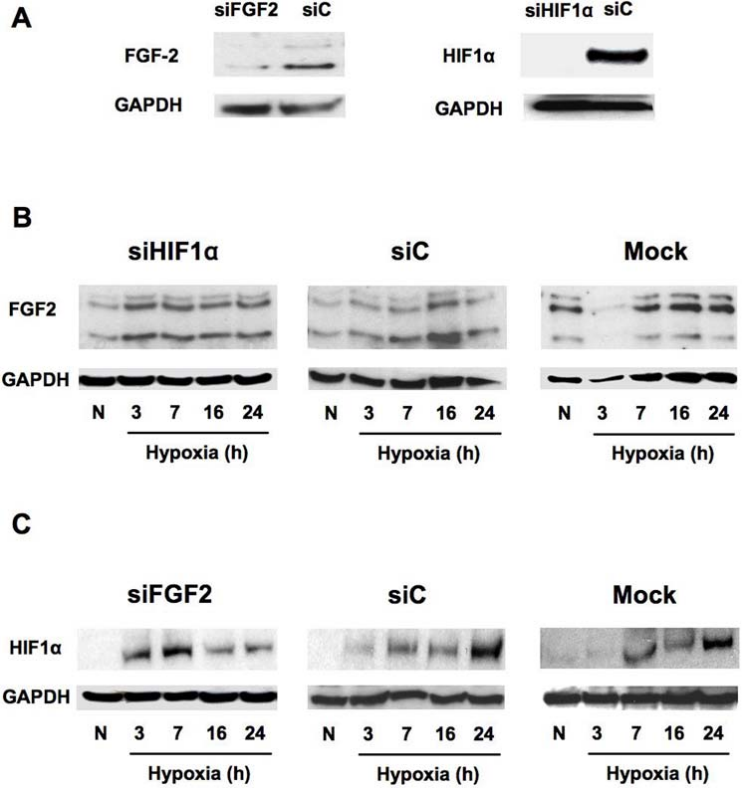
Crosstalk between FGF2 and HIF-1α. A. FGF2 and HIF-1α gene silencing using targeted-siRNA. 911 cells were transfected with FGF2 (left) or HIF-1α (right) targeted siRNA (siFGF2 or siHIF-1α, respectively). Protein contents were measured in normoxic (FGF2) and hypoxic (HIF-1α) cells 48 h after transfection. SiC corresponds to a control scrambled siRNA. B. Effect of FGF2 gene silencing on HIF-1α accumulation in 911 cells cultivated in normoxic (N) and hypoxic conditions. C. Effect of HIF-1α gene silencing on FGF2 accumulation in 911 cells cultivated in normoxic (N) and hypoxic conditions.

The decrease in FGF2 mRNA expression observed in ischemic skin and in late hypoxia in vitro, concomitant with the appearance of HIF-1α, suggested that FGF2 expression is not activated by the classical HIF-1α transactivating effect. To know whether FGF2 induction was HIF-dependent or not, 911 cells were treated with siRNA against HIF-1α prior to hypoxia (Fig. 5). Strikingly, HIF-1α knock down resulted in FGF2 induction after 3 h of hypoxia, and abrogated the peak of FGF2 induction observed at 16 h in the control siRNA- and mock-transfected cells (Fig. 5B). This experiment suggested that the small amount of HIF-1α expressed at 3 h (see Fig. 4A) is able to repress expression of FGF2. Interestingly, 3 h is also the time at which FGF2 mRNA levels increase (Fig. 4B), suggesting that an early effect of HIF-1α on FGF2 expression is the translational repression of FGF2 mRNA. This translational blockade would be relieved in the presence of large amounts of HIF-1α, which accumulated by 16 h.

In our *in vivo* experiments, we observed that FGF2 induction begins 6 h after surgery, prior to HIF-1α accumulation, which is detected only after 24 h. This suggests that FGF2 signaling may act on HIF-1α induction, as shown in recent reports [27], [28]. In order to establish whether FGF2 might have an influence on HIF-1α accumulation during hypoxia, FGF2 was knocked down in 911 cells by siRNA treatment and protein expression was measured. The results showed that FGF2 knock down generates HIF-1α accumulation during early hypoxia, followed by a strong decrease of HIF-1α protein in later stages of hypoxia (Fig. 5C). Thus while the small amount of FGF2 that is present at the 3 h time point is able to prevent HIF-1α accumulation, the larger amount of FGF2, which is induced at later times, has a positive effect on HIF accumulation.

These data revealed a complex crosstalk between FGF2 and HIF-1α during the hypoxic response. FGF2 and HIF seem to regulate each other in two successive feedback loops: in early hypoxia (3 h), FGF2 and HIF-1α, present in small amounts, behave as mutual inhibitors in a negative regulatory loop, whereas in late hypoxia (16 h) they amplify each others expression in a positive regulatory loop.

## Discussion

In this study we demonstrate that FGF2 expression is up-regulated in response to ischemia *in vivo* as well as under hypoxic conditions *in vitro*. Strikingly, this up-regulation does not occur at the mRNA level but at the protein level, revealing new mechanistic aspects of the hypoxic response. Our data show that hypoxia generates a strong increase of FGF2 IRES-mediated translation both *in vivo* and *in vitro*, when cap-dependent translation is blocked. This suggests that FGF2 induction during hypoxia occurs by a translational mechanism, providing an important pathophysiological function to the IRES present in the FGF2 mRNA. In addition, our data reveal that FGF2 interacts with HIF-1α in a very unusual crosstalk, different from the classical angiogenic growth factor induction by HIF-1α. FGF2 and HIF-1α regulate each others expression by a negative feedback loop in early hypoxia, followed by a positive feedback loop in late hypoxia.

A striking feature of our data is that induction of FGF2 protein is concomitant with a decrease in FGF2 mRNA, *in vivo*. In addition, FGF2 protein induction *in vitro* correlates with a decrease in mRNA accumulation. This indicates that FGF2, in contrast to classical HIF-1α targets, can be induced by hypoxia without transcriptional activation, despite the putative hypoxia responsive element (HRE) recently characterized in the FGF2 gene promoter [29]. This HRE, whose activity has been described in pulmonary arterial smooth muscle cells, is clearly not involved in FGF2 induction in ischemic skin at the time points used in our study. However, in human retinoblasts 911, FGF2 mRNA levels increase during early hypoxia (3 h), then decrease in late hypoxia (7 h to 24 h), suggesting that, in these cells, FGF2 would be subjected to two steps of regulation: transcriptional induction would occur first (where the mRNAs remain translationally silent), and would be followed by FGF2 protein induction concomitant with a decrease in mRNA levels. Thus hypoxia may induce FGF2 by distinct mechanisms, probably depending upon the cell type or tissue.

An unexpected finding concerns the decrease in FGF2 mRNA levels that accompany the increase in FGF2 protein levels. Such an inverse correlation between FGF2 protein and mRNA levels has been previously reported and might be mediated by an instability element identified within the FGF2 mRNA 3′untranslated region [30], [31]. Efficient translation of FGF2 transcripts could activate their degradation. Such an observation questions the numerous studies based on transcriptome analyses, which may lead to a misinterpretation of gene expression levels.

Our study shows that FGF2 increases at the protein level in response to hypoxia. Such a protein accumulation may be due either to protein stabilization, activation of FGF2 mRNA translation or both. Several previous reports from the literature have reported that FGF2 expression is controlled at the translational level, whereas no information is available about the FGF2 protein degradation process [32], [33]. To analyze FGF2 protein accumulation during hypoxia, we used the siRNA knock down approach (Fig. 4C). These data, showing a disappearance of FGF2 during late hypoxia and in the absence of mRNA translation, allows us to rule out an increase in FGF2 protein stability by hypoxia. Furthermore we show that FGF2 IRES-dependent translation is enhanced under hypoxic conditions *in vivo* as well as *in vitro*, whereas 4E-BP hypophophorylation in ischemic skin indicates a repression of cap-dependent translation (Fig. 3C). The inhibition of cap-dependent translation by hypoxia *in vitro* has been previously described in several other reports [34]–[36]. Furthermore our results agree with a previous study showing activation of the FGF2 IRES in a murine ischemic hind leg model [7]. Taken together, these data indicate that FGF2 is induced in late hypoxia by a translational event most probably involving the IRES. Our study shows that IRES-dependent regulation in a bicistronic mRNA context is much more sensitive to hypoxia *in vivo* than *in vitro*: the IRES activity is up-regulated 40 times in mouse skin (Fig. 3A), versus only 4 times in hypoxic 911 cells (Fig. 4D). This points out the importance of studying such regulations in pathophysiological situations *in vivo*, and suggest that *in vitro* studies may underestimate the physiological regulation of IRES activity.

A very amazing result is that expression of the LucR cistron, which is expected to be cap-dependent and to decrease during ischemia, is activated 24 h after surgery, concomitant with IRES-dependent activation of LucF (Fig. 3A). The decrease of bicistronic mRNA levels under ischemic conditions, shown in Fig. 3B, indicates that such an activation does not result from an induction of mRNA transcription. While one might argue that cap-dependent translation is not repressed under our conditions, there exist numerous reports describing the blockade of cap-dependent translation by stress and we clearly demonstrate that 4E-BP is hypophosphorylated in our system (Fig. 3C). Hypophosphorylated 4E-BP sequesters the cap-binding factor eIF-4E and reflects the repression of cap-dependent translation [25], [34]. One possible explanation for the observed increase in LucR is provided by a recent paper that reports that the IRES in a bicistronic construct stimulates translation of the upstream cistron [37]. The authors conclude that whenever eIF-4F has been captured to a bicistronic mRNA by binding to a picornavirus IRES via its eIF-4G moiety, it can be provided in cis to the 5′ end of the mRNA and stimulate translation initiation. An IRES-dependent activator effect of the first cistron has also been observed with a cellular IRES in a recent report using C2C12 myoblasts and a bicistronic construct containing the FGF1 IRES [38].

However, the model proposed above can only partially explain our data, as functional eIF-4F (the protein complex containing the cap-binding protein eIF-4E) is present in low amounts in our system where eIF-4E is sequestered by 4E-BP. Another hypothesis is based on a hypoxia-dependent formation of stress granules (SGs) that sequester mRNAs in a translationally silenced state [39], [40]. SGs contain the majority of polyadenylated mRNAs that are subject to stress-induced translational arrest but exclude mRNAs encoding stress-induced proteins. Thus we can hypothesize that mRNAs containing the FGF2 IRES that are induced by hypoxia might be excluded from stress granules. The presence of the IRES would thus generate an active recruitment of the bicistronic mRNA into polysomes and result in active translation of the first cistron. Further investigation will be required in order to resolve this issue. At this time we can only conclude that one must be cautious when calculating IRES activity as the ratio between LucF and LucR activities, as it may in fact be an underestimate of the actual IRES activity.

The positive amplification feedback between FGF2 and HIF-1α observed during late hypoxia in our study is supported by recent reports. One such study has uncovered the existence of an HIF-1α/FGF2 autocrine loop, which induces an angiogenic response in human endothelial cells (HUVECs) [27]. Using FGF2 neutralizing antibodies, the authors show that FGF2 is required for late but not early induction of HIF-1α. This autocrine effect is specific to FGF2, whereas antibodies against VEGF, IGF-1 or PDGF-BB do not influence HIF-1α induction. The same report shows using a HIF-1α siRNA, that HIF-1α is required for hypoxic induction of FGF2 mRNA and for FGF2 protein secretion in HUVEC culture supernatant [27]. Another report has shown that HIF-1α up-regulation by hypoxia in myoblasts requires FGF2 and heparane-sulfate containing low-affinity FGF2 binding sites [28].

More unexpected is the negative feedback uncovered here between FGF2 and HIF-1α during early hypoxia: Fig. 5B and 5C clearly show that HIF-1α siRNA up-regulates FGF2 protein whereas FGF2 siRNA up-regulates HIF-1α after 3 h of hypoxia. This negative feedback occurs when both FGF2 and HIF-1α are present in low amounts, suggesting that it could occur beneath a given threshold of the two proteins and that the amplification loop might start when this threshold is exceeded. The consequence of this negative feedback is a delay of the stress response. Interestingly, a previous report revealed that during hypoxia, HIF-1 regulated transcripts are translationally silenced by sequestration into stress granules, resulting in a down-regulation of HIF signaling [40]. The translational inhibition is relieved later upon reoxygenation, when the cell becomes able to rapidly translate the once-sequestered HIF-1 regulated transcripts, allowing it to recover from the hypoxic shock. In our data, the presence of untranslated FGF2 mRNA after 3 h of hypoxic stress (Fig. 4B) could be explained by its sequestration in stress granules. Then IRES activation after 4 h (Fig. 4D) would allow the FGF2 mRNA to be excluded from stress granules, resulting in FGF2 protein expression.

With respect to the effect of FGF2 on HIF-1α expression, the activator effect has been shown to occur by an autocrine mechanism, implicating an involvement of FGF2 secretion and FGF2 receptor signaling [27]. However FGF2 is expressed as five isoforms with different localizations and functions [12], [15], [41]. The high molecular weight isoforms, which are not secreted, remain nuclear and act by an intracrine mechanism that might be responsible for the early negative effect of FGF2 on HIF-1α expression during hypoxia [42].

Taken together, our data underline the importance of translational regulation and the unique role of FGF2 in its crosstalk with HIF-1α in the hypoxic response. However they also point to the complexity of the cellular response to hypoxia, and incite us to decipher more deeply the mechanisms governing the dialog between HIF-1α and FGF2 to find new molecular triggers of tumour angiogenesis.

## Materials and Methods

### Mice

Transgenic mice used in this study have been already described [18]. Briefly, mice carry transgenes coding for two luciferase genes, Renilla luciferase (LucR) and Firefly luciferase (LucF), both controlled by the cytomegalovirus promoter (CMV) and separated by the FGF2 IRES. Transgenic mice were bred in our animal facilities and acclimatized to experimental conditions for at least one week before surgery. All procedures were performed in accordance with the recommendations of the European Accreditation of Laboratory Animal Care Institute.

### Ischemia model

A standardized model of soft tissue ischemia was modified from a surgical procedure previously described by Ceradini et al [26]. After general anaesthesia with a mixture of ketamine (100 mg kg^−1^) and xylazine (10 mg kg^−1^), the backs of eight week old female transgenic homozygote mice were shaved and a U-shaped peninsular incision (2.5 cm in length 1.25 in width) was created on the dorsal skin, including the epidermis, dermis and platysma muscle. The two vascular pedicles arising from the lateral thoracic arteries were systematically sectioned. The skin was then put in place and sutured with 5.0 nylon sutures (Silkam®, B. Braun, France). 6, 24, 48 or 72 hours after surgery, mice were sacrificed. The proximal part of the skin flap was removed at different times post-surgery and frozen.

### Cell culture

The 911 human embryonic retinoblasts obtained from the European Collection of Cell Culture were stably transfected with the already described pCRFL (911-FGF2) or pCREL (911-EMCV) plasmids (Lourenco-Dias, PhD thesis, 2006).

For hypoxic treatment, cells were plated under normoxic conditions and grown for 16–20 h and then placed into a hypoxic chamber (Binder, Tuttlingen, Germany) stabilized at 1%O2, 5%CO2 at 37°C. The small interfering RNAs were purchased from Dharmacon siGENOME® SMARTpool® HIF-1α siRNA, siGENOME® SMARTpool® FGF-2 siRNA and siGENOME® nontargeting siRNA. 911 cells were transfected with 10 nM siRNA with the lipofectamine 2000 transfection reagent (Invitrogen). Cells were put under hypoxic conditions 48 h after transfection.

### Luciferase Activity Assay

Frozen skin was homogenized in 250 µl of passive lysis buffer (Promega). LucR and LucF activities were measured using the Dual Luciferase kit from Promega.

### Western Blot analysis

Frozen skins placed in Lysing Matrix A tube (Q-BIOgene, Carlsbad, CA) were homogenized in 500 µl of SDS 2% lysis buffer at 95°C using FastPrep® Sample Preparation system. FGF2 protein was immunodetected as previously described [22]. HIF-1α protein was immunodetected with a mouse monoclonal antibody (R&D systems), 4E-BP with a rabbit polyclonal antibody (Cell Signaling) and GAPDH with a mouse monoclonal antibody (Santa Cruz Inc.).

### Quantitative real-time RT-PCR

Total RNA was extracted from frozen skin using the RNeasy Mini Kit (Qiagen, France). Reverse Transcription was performed with the High capacity cDNA Archive Kit (Applied Biosystems, Foster City, CA). As an internal control, the quantification of ribosomal L8 RNA was used. Specific primers and probes are:

- primer forward mFGF-2 5′CACCAGGCCACTTCAAGGA3′, primer reverse mFGF-2 5′ GATGGATGCGCAGGAAGAA3′.
- primer forward LucR1 5′AGCCAGTAGCGCGGTGTATT3′, primer reverse LucR1 5′ CAAGTAACCTATAAGAACCATTACCAGATTT3′, probe LucR1, 5′CCAGACCTTATTGGTATGGGCAAATCAGG3′;
- primer forward LucR2 5′ GCTTATCTACGTGCAAGTGATGATTT3′, primer reverse LucR2 5′ CCTTCAACAATAGCATTGGAAAAGA3′, probe LucR2, 5′ CCAAAAATGTTTATTGAATCGGACCCAGGA3′;
- primer forward LucF 5′ TTCCATCTTCCAGGGATACGA3′, primer reverse LucF 5′ATCATCCCCCTCGGGTGTA3′, probe LucF, 5′TGGGCTCACTGAGACTACATCAGCTATTCTGA3′.

Quantitative PCR was performed as previously described [22].

### Statistical analysis

Results are expressed as mean±SEM. ANOVA and Student t-test were used to determine statistically significant differences. Statistical analysis was performed with the software Prism (Prism®, GraphPad).
